# Infective endocarditis at a tertiary-care hospital in China

**DOI:** 10.1186/s13019-020-01183-2

**Published:** 2020-06-10

**Authors:** Lulu Ma, Ying Ge, Haobo Ma, Bo Zhu, Qi Miao

**Affiliations:** 1 Department of Anesthesiology, Peking Union Medical College Hospital, Chinese Academy of Medical Sciences and Peking Union Medical College, Beijing, 100730 China; 2 Department of Infectious Diseases, Peking Union Medical College Hospital, Chinese Academy of Medical Sciences and Peking Union Medical College, Beijing, 100730 China; 3 Department of Anesthesia, Critical Care and Pain Medicine, Beth Isreal Deaconess Medical Center, Boston, MA 02215 USA; 4 Department of Cardiac Surgery, Peking Union Medical College Hospital, Chinese Academy of Medical Sciences and Peking Union Medical College, Beijing, 100730 China

**Keywords:** Infective endocarditis, In-hospital mortality, Viridans group streptococci

## Abstract

**Background:**

The aim of this study was to describe the clinical features and outcome of infective endocarditis at a general hospital in China and to identify the risk factors associated with in-hospital mortality.

**Methods:**

A retrospective study was conducted and all patients diagnosed with definite or possible infective endocarditis between January 2013 and June 2018 according to the modified Duke criteria were included.

**Results:**

A total of 381 patients were included. The mean age was 46 years old and 66.9% patients were male patients. Community acquired IE was the most common type of infective endocarditis and Viridans Group Streptococci (37.5%) was still the most common causative pathogen. The microbial etiology of infective endocarditis varied with location of acquisition. 97 (25.5%) patients had culture-negative infective endocarditis. Vegetations were detected in 85% patients and mitral valve was the most common involved valve. Operations were performed in 72.7% patients and in-hospital mortality rate was 8.4%. The risk factors of in-hospital mortality were age old than 70 years old, heart failure, stroke and medical therapy.

**Conclusions:**

Older age, heart failure, stroke and medical therapy were risk factors of in-hospital mortality. Infective endocarditis, were mainly caused by Viridans Group Streptococci, characterized by younger patients and lower mortality rate in China.

## Background

Infective endocarditis (IE) is an uncommon infective disease. Heart failure and embolisms of vegetations to the brain and other organs are life-threatening complications. Despite advances in diagnosis, medical and surgical treatment, the variability of disease presentation is a challenge to clinicians and the mortality remains high. The in-hospital mortality rate is 15–20% [1, 2] and the one-year mortality is 40% [2–4]. Early diagnosis, antibiotic therapy or surgery is necessary.

Few studies have been performed to describe the characteristics of IE in China, the aim of this study was to describe the clinical characteristics of IE in a single center in China and to identify the risk factors associated with in-hospital mortality.

## Methods

A retrospective study was conducted at Peking Union Medical College Hospital. This study has been granted an exemption from requiring ethics approval according to “The ethics committee of Peking Union Medical College Hospital”.

Patients who were diagnosed with definite or possible IE according to modified Duke criteria between January 2013 and June 2018 were included. Medical records were reviewed and clinical information which included age, gender, comorbidities, clinical signs and symptoms, possible source of infections, previous history of IE, valves involved, laboratory and echocardiographic findings and treatment was retrospectively collected.

Community –acquired IE was that diagnosed at the time of admission or ≤ 48 h of admission. Health care associated IE included nosocomial and non-noscomial health care-associated IE. Nosocomial IE was defined as IE developing in patients hospitalized for more than 48 h without manifestations of IE before admission. Non-nosocomial health care-associated IE was defined if signs or symptoms of IE developed before hospitalization in patients with extensive health care contact, including (1) intravenous therapy, wound care or specialized nursing care at home within the 30 days before the onset of IE, (2) visiting a hospital or hemodialysis clinic or receiving intravenous chemotherapy within the 30 days before the onset of IE, (3) hospitalization in an acute care hospital for ≥2 days in the 90 days before the onset of IE, (4) staying in a nursing home or long-term care facility [5].

Systemic use of corticosteroids or chemotherapy for more than 1 month was defined as immunosuppressive therapy. Cancer was defined as those with active disease or on chemotherapy. Immunological phenomena included glomerulonephritis, Osler’s nodes, Roth spots and rheumatoid factor positivity. Vascular phenomena included major arterial emboli, septic pulmonary infarcts, mycotic aneurysm, intracranial hemorrhage, conjunctival hemorrhages and Janeway’s lesions. Antimicrobial therapy was based on either the American Heart Association Guidelines [6] or the European Society of Cardiology guidelines [7]. Antibiotics started as soon as the results of blood cultures had been acquired, and 4–6 weeks was usually necessary. For acute ill patients with suspected IE, empiric therapy which covered staphylococci, streptococci and enterococci would initiate first. Once the pathogen was identified, the antibiotic treatment will be modified according to the pathogen, antimicrobial susceptibility pattern, severity of infection and also the type of IE. The indications of surgical intervention in our center included heart failure, recurrent embolic event, vegetation size (> 10 mm), persistent bacteremia, paravalvular complications, prosthetic valve endocarditis, and microorganism of fungi or high resistant organisms. In-hospital mortality was defined as death occurring during hospitalization for IE.

Statistical analyses were performed with SPSS software, version 19.0 (SPSS Inc., Chicago, Illinois). Quantitative data was shown as mean + SD. Categorical variables were summarized using frequency and percentages. Student t test was used to compare variables between two groups. Chi-square test was used to compare categorical variables. Logistic regression analysis was performed to examine correlation of variables to in-hospital mortality. *P* value < 0.05 (2 -sided) was considered statistically significant.

## Results

Three hundred eighty-one patients who were diagnosed with definite IE or possible IE were included. Community acquired IE (87.4%) was the most common type of IE, followed by non-nosocomial health care associated IE (6.6%) and nosocomial health care associated IE (6.0%) (Table 1). Native valve involvement was detected in 352(92.4%) patients, followed by prosthetic valve related IE (6.8%) and cardiac device –relate IE (0.8%). There were 291 cases (75.8%) of left-side IE and 54 cases (14.1%) of right-side IE.

**Table 1:** Type of IE and microbiologic etiology of IE

**Type of IE**	
Community-acquired	333 (87.4%)
Nosocomial health care associated	23 (6.0%)
Non-nosocomial health care associated	25 (6.6%)
**Valve involved**	
Native valve involvement	352 (92.4%)
Prosthetic valve involvement	26 (6.8%)
Cardiac device-related IE	3 (0.8%)

**Fig. 1:**
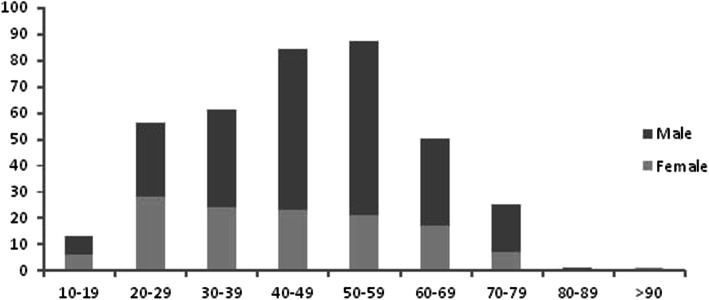
Age and sex distributions of patients

The demographic characteristics were summarized in Table 2. The mean age was 46 years old and 255 patients (66.9%) were male. Age and sex distributions of patients were shown in Fig. 1.

**Table 2:** Demographic characteristics and predisposing conditions in 381 patients

Variables	Total	Community acquired (*n* = 333)	Non-nosocomial (*n* = 25)	Nosocomial (*n* = 23)	P
Age, years	46 + 16	46 + 16	46 + 17	52 + 14	0.275
Male	255 (66.9%)	217 (65.2%)	20 (80%)	18 (78.3%)	0.155
Predisposing medical disease					
Degenerative valve disease	82 (21.5%)	78 (23.4%)	4 (16%)	0 (0%)	0.024
Hypertension	77 (20.2%)	61 (18.3%)	6 (24%)	10 (43.5%)	0.013
Congenital heart disease	68 (17.8%)	63 (18.9%)	3 (12%)	2 (8.7%)	0.344
Diabetes mellitus	34 (8.9%)	27 (8.1%)	3 (12%)	4 (17.4%)	0.274
Alcohol abuse	26 (6.8%)	21 (6.3%)	4 (16%)	1 (4.3%)	0.159
Rheumatic heart disease	23 (6.0%)	20 (6.0%)	1 (4%)	2 (8.7%)	0.791
Immunocompromised state	22 (5.8%)	10 (3.0%)	3 (12%)	9 (39.1%)	< 0.001
Coronary heart disease	20 (5.2%)	17 (5.1%)	2 (8%)	1 (4.3%)	0.806
Cancer	13 (3.4%)	10 (3.0%)	1 (4%)	2 (8.7%)	0.342
History of IE	10 (2.6%)	9 (2.7%)	1 (4%)	0 (0%)	0.666
Hypertrophic cardiomyopathy	7 (1.8%)	7 (2.1%)	0 (0%)	0 (0%)	0.598
Dialysis	5 (1.3%)	0 (0%)	4 (16%)	1 (4.3%)	< 0.001
Pregnancy	2 (0.5%)	2 (0.6%)	0 (0%)	0 (0%)	0.865

Degenerative valve disease and congenital heart disease were the common underlying cardiac diseases. Bicuspid aortic valve (50 cases, 13.1%) and mitral valve prolapse were (28 cases, 7.3%) were the most common degenerative valve disease. Congenital heart disease was found in 68 cases, among which there were 29 cases of ventricular septal defect and 21 cases of patent ductus arteriosus.

Twenty-seven patients (7.1%) had dental procedure before the onset of the symptoms and 12 of them had prophylactic antibiotic treatment. There are 38 (10.0%) postsurgical cases, with 13 cases of cardiac surgeries and 25 cases of non-cardiac surgeries. 31 (86.5%) of them had antibiotics after operations. Three patients had a history of trauma and intravenous drug use (1 case) was uncommon in our cohort.

There was no difference in age and sex distribution among community acquired IE, non-nosocomial health care associated IE and nosocomial health care associated IE. The proportion of degenerative valve disease was higher in community acquired IE (*P* = 0.024), immunocompromised state was more common in nosocomial IE (*P* < 0.001), while dialysis was more common in non-nosocomial IE (*P* < 0.001).

Blood cultures were taken to determine the causative microorganism. Serologic tests and valve cultures were also performed in a small portion of patients. 97 (25.5%) patients had negative culture result. Gram positive organisms were the most common causative microorganism. The microbial etiology of IE varied with location of acquisition, with a higher proportion of Viridans group Streptococci among patients with community acquired infection. (Table 3).

**Table 3:** Causative pathogens and acquisition of infective endocarditis

Causative pathogen	Total (*n* = 381)	Community acquired (*n* = 333)	Non-nosocomial I (*n* = 25)	Nosocomial (*n* = 23)	P
Gram-positive bacteria					
Viridans group streptococci	143 (37.5%)	134 (40.2%)	8 (32%)	1 (4.3%)	0.002
*Staphylococcus aureus*	30 (7.9%)	21 (6.3%)	5 (20%)	4 (17.3%)	0.011
MRSA	10 (2.6%)	8 (2.4%)	1 (4%)	1 (4.3%)	0.772
MSSA	20 (5.2%)	13 (3.9%)	4 (16%)	3 (13.0%)	0.007
CNS	12 (3.1%)	8 (2.4%)	2 (8%)	2 (8.7%)	0.088
*Enterococcus faecalis*	11 (2.9%)	8 (2.4%)	3 (12%)	0 (0%)	0.015
*Streptococcus bovis*	6 (1.6%)	6 (1.8%)	0 (0%)	0 (0%)	0.644
Other	16 (4.2%)	14 (4.2%)	2 (8%)	0 (0%)	0.386
Gram-negative rods	21 (5.5%)	16 (4.8%)	0 (0%)	5 (21.7%)	0.001
HAECK	12 (3.1%)	11 (3.3%)	0 (0%)	1 (4.3%)	0.623
Rickettsia	4 (1.0%)	4 (1.2%)	0 (0%)	0 (0%)	0.747
TB	1 (0.26%)	1 (0.3%)	0 (0%)	0 (0%)	0.930
Fungi	6 (1.6%)	3 (0.9%)	1 (4%)	2 (8.7%)	0.009
Others	12 (3.1%)	12 (3.6%)	0 (0%)	0 (0%)	0.409
Polymicrobial	10 (2.6%)	7 (2.1%)	2 (8%)	1 (4.3%)	0.178
Culture negative	97 (25.5%)	88 (26.4%)	2 (8%)	7 (30.4%)	0.106

Fever was the most common clinical manifestation. Heart failure was observed in 70(18.4%) patients. Vascular phenomena (42.8%) were more common than immunological phenomena (40.0%). Abscess, which included cerebral, splenic, pulmonary and vertebral abscess was found in 40(10.5%) patients. (Table 4).

**Table 4:** Clinical manifestation and ultrasonic findings in 381 patients

Clinical manifestation	
First clinical manifestation< 1 month	133 (34.9%)
Fever	367 (96.3%)
New murmur	157 (41.2%)
Vascular phenomena	163 (42.8%)
Stoke	88 (23.1%)
Heart failure	70 (18.4%)
Abscess	40 (10.5%)
Anemia	259 (68%)
Immunological phenomena	118 (31%)
Vegetation	325 (85%)
Involved valve	
Aortic valve	127 (33.3%)
Mitral valve	139 (36.5%)
Tricuspid valve	32 (8.4%)
Pulmonary valve	14 (3.7%)
Others	7 (1.8%)
Multi	40 (10.5%)
Intra-cardiac implantation	2 (0.5%)
New severe mitral regurgitation	71 (18.6%)
New severe aortic regurgitation	77 (20.2%)
Paravalvular complication	47 (12.3%)
Paravalvular fistula	13 (3.4%)
Abscess	34 (8.9%)

All patients had echocardiography and 85% had echocardiographic evidence of vegetation. 57(15%) patients had both transthoracic and transesophageal echocardiography. Mitral valve was the most common involved (36.5%), followed by the aortic valve (33.3%). New severe mitral regurgitation and aortic regurgitation were detected in 71(18.6%) and 77(20.2%) patients, respectively. Paravascular complications, which included paravascular fistula and abscess were observed in 47 patients.

Operation was performed in 277(72.7%) patients, and 29 patients had emergent operations. Among patients who did not have operation, 53 patients recovered after medical treatment. Forty-seven patients had surgical indications, and patients’ refuse (34 patients), cerebral infarction (10 patients), surgical difficult (2 patients), hemodynamic instability (2 cases) and other medical causes (2 patients) are reasons for no surgery. In-hospital mortality was 8.4% (32 patients). The in-hospital mortality rate in community acquired IE, non-nosocomial health care associated IE and nosocomial health care associated IE was 6.3, 12 and 34.8% respectively. Multivariable regression analysis showed that age older than 70 years old, heart failure, stroke and medical therapy are risk factors of in-hospital mortality. (Table 5).

**Table 5:** Predictors of in-hospital mortality of IE patients

	Survisors (*n* = 349)	Death (*n* = 32)	*p*	OR	Multivariable regression	
Age (older than 70)	19	8	0.002	4.583 (1.861, 11.285)	3.887 (1.072,14.096)	0.039
Sex (Male/Female)	230/119	25/7	0.175	0.541 (0.227,1.288)		
Rheumatic heart disease	21	2	1.000	1.041 (0.233,4.656)		
Degenerative valve disease	49	1	0.100	0.197 (0.026,1.480)		
Congenital heart disease	66	2	0.090	0.286 (0.067,1.226)		
Hypertrophy cardiomyopathy	7	1	0.508	1.576 (0.188,13.226)		
History of IE	9	1	0.589	1.219 (0.149,9.936)		
Hypertension	64	13	0.005	3.047 (1.431,6.488)		
DM	28	6	0.053	2.646 (1.005,6.965)		
Immunocompromised state	15	7	0.001	6.235 (2.328,16.694)		
dialysis	2	3	0.005	17.948 (2.882,111.761)		
cancer	9	4	0.017	5.397 (1.563,18.635)		
Health care associated IE	37	11	0.001	4.417 (1.974,9.881)		
Native valve involvement	322	30	1.000	1.28 (0.285,5.549)		
Prosthetic valve involvement	24	2	1.000	1.108 (0.250,4.916)		
Cardiac device-related IE	3	0	1.000	0.991 (0.982,1.001)		
Fever	336	31	1.000	0.834 (0.106,6.587)		
New murmur	142	15	0.574	1.286 (0.622,2.660)		
Heart failure	52	18	0.000	7.343 (3.441,15.671)	7.955 (2.966,21.338)	0.000
Vascular phenomena	144	19	0.061	2.081 (0.996,4.348)		
stroke	71	17	0.000	4.212 (2.009,8.830)	4.190 (1.669,10.519)	0.002
Immunological phenomena	113	5	0.070	0.387 (0.145,1.031)		
Viridans group streptococci	136	7	0.059	0.43990.185,1.042)		
Staphylococcus aureus						
MRSA	9	1	0.589	1.219 (0.149,9.936)		
MSSA	18	2	0.680	1.226 (0.271,5.538)		
CNS	11	1	1.000	0.991 (0.124,7.933)		
Enterococcus faecalis	10	1	1.000	1.094 (0.135,8.826)		
Streptococcus bovis	5	1	0.411	0.451 (0.051,3.979)		
HAECK	12	0	0.610	0.966 (0.947,0.985)		
Rickettsia	4	0	1.000	0.989 (0.977,1.000)		
TB	1	0	1.000	0.997 (0.992,1.003)		
Fungi	3	3	0.009	11.931 (2.304,61.787)		
Polymicrobial	8	2	0.201	2.842 (0.577,13.988)		
Vegetation	298	27	0.798	0.924 (0.340,2.511)		
Aortic valve	117	10	0.847	0.901 (0.413,1.966)		
Mitral valve	127	12	1.000	1.049 (0.496,2.216)		
Tricuspid valve	31	1	0.500	0.331 (0.044,2.507)		
Pulmonary valve	14	0	0.618	0.960 (0.940,0.981)		
Multi valve involvement	34	6	0.128	2.138 (0.822,5.559)		
New severe mitral regurgitation	48	7	0.198	1.756 (0.720,4.283)		
New severe aortic regurgitation	59	6	0.806	1.134 (0.447,2.877)		
Medical therapy	81	23	0.000	0.118 (0.053,0.266)	6.854 (2.632,17.848)	0.000

## Discussion

This retrospective study described the epidemiological and clinical features of IE in a single center in China. The average age was 46 years old and Viridans group streptococci was still the most common pathogen. Surgical therapy was performed 72.7% patients. The risk factors of in-hospital mortality included age older than 70 years old, heart failure, stroke and medical therapy.

The mean age of patients in our cohort was 46 years old, which was younger than that in developed countries ranging from to 52.9 to 69.1 years old [8–10]. But the mean age in our cohort was older than another study conducted in China from 1998 to 2009 [11]. The reasons for increasing trend of age of IE patients in China were related to decreased rate of rheumatic heart disease and congenital heart disease, and increased rate of degenerative valve disease.

Blood culture was still the major criteria for infective endocarditis. Viridans group streptococci were the most common pathogen in our cohort, which was consistent with previous report in china, Laos and Japan [10–13]. However, *Staphylococcus aureus* was the most common causative pathogen in developed countries [8, 9, 14]. We also noticed the different causative pathogen varied with location of acquisition and *Staphylococcus aureus* was more common in health care associated IE. The reasons of low frequency of staphylococcus infection in our study were as follows. First, the majority of IE in our cohort was community-acquired IE and native valves were the most frequently involved valves. The prevalence of risk factors for *Staphylococcus aureus*-associated IE (for example, drug abuse, health care contact and invasive procedures) [9, 12] in our study was low. Second, Viridans group Streptococci was the common pathogen in patients with poor dental health. It reflects the poor dental hygiene in Chinese population [12].

Culture-negative IE remains a challenge in clinic. The possible reasons included exposure to slow-growing bacterium, non-infective endocarditis, administration of antibiotics before blood culture, right-side IE, cardiac device-related IE or IE associated with intravascular device or foreign bodies [15]. In our study, the incidence of negative blood culture was 25.5%, which was higher than previous reports [8–10]. Previous antibiotic administration contributed to the higher incidence of negative blood culture in our study. Metagenomic analysis has recently been successfully used in detecting pathogens in culture-negative infective endocarditis [16, 17], however more researches are required to prove its potential role in diagnosing culture-negative infective endocarditis.

Vegetations were detected in 85% patients in our study, which was similar to previous reports [9, 10]. Transthoracic or transesophageal echocardiography is the mainstay in diagnosis of IE. The possibilities of no detection of vegetations were as following. First, the size of vegetation may be too small to de detected, and transesophageal echocardiography was not performed in all patients. Second, vegetations may disappear due to embolism. Third, antibiotic therapy had started before echocardiography.

Surgical therapy was performed in 72.7% patients which was higher than previous reports [8–10]. The indications of surgical intervention in our center were consistent with the recommendations from American Heart Association [6] and ESC guideline [7]. The majority of patients in our study were referred for surgical therapy from other hospitals was the main reason for this high percentage of patients being operated. And the average age of our cohort was younger than that in developed countries, and elderly patients with IE were less likely to have surgical treatment due to higher risk [18].

The overall in-hospital mortality rate was 8.4%, which was lower than previous reports [8–10]. However, the in-hospital mortality rate of non-nosocomial health care associated IE and nosocomial health care associated IE was 12 and 34.8% respectively. Patients’ characteristics, complications of IE, microorganism and the echocardiographic findings can all influence the prognosis [7]. The possible reasons which contributed the lower in-hospital mortality in our study were as followings. First, the average age of our study is younger than previous studies. And the mortality rate in elderly patients with IE was high [18]. Second, the main pathogen was Viridans group Streptococci in our cohort, while the proportions of *Staphylococcus aureus* and Fungi were low. Streptococcal IE was associated with a decreased risk of in-hospital mortality [9]. Third, the majority of IE was community acquired IE in our study, while health-care related IE was the independent predictors of in-hospital mortality [8]. Besides these, native valve was the most common involved. Patients undergoing surgical treatment for prosthetic valve endocarditis had a higher 30-day and 1-year mortality, when compared to native valve endocarditis [19]. The proportion of congenital heart disease, which was associated with better outcome than other forms of IE [7], was higher in our study [9, 10]. The last but not the least was the high rate of surgical treatment. Better prognosis had been reported in IE patients with a higher rate of valvular surgery [20, 21].

Multivariable regression analysis showed older age, heart failure, stroke and medical therapy were independent risk factors of in-hospital mortality. Among these factors, heart failure had the highest odds ratio. Some studies have also confirmed that these factors were associated with in hospital mortality [8, 22–26]. And perivascular abscess [19], diabetes mellitus [27, 28], or acute renal failure [26, 29] have also been reported to be the risk factors of high mortality. Interestingly, medical therapy was associated with higher in-hospital mortality, which suggested the protective role of surgical treatment. However, whether the surgical treatment was associated with better outcome or the higher proportion of surgical treatment contributed to the lower in-hospital mortality in our study still needs further research.

There were several limitations of this study. First, this was a retrospective study in a single center, the selection bias was unavoidable and the accuracy of data was limited. Second, the limited number of patients made the impossibility of generalization of our results. Third, this was a retrospective study, and long-term follow-up was lacking in the majority of patients.

## Conclusions

Our study demonstrates that IE in China remains as a clinical challenge. Viridans group streptococci was still the most common pathogen and community acquired infection was the most acquisition of IE. Older age, heart failure, stroke and medical therapy were independent risk factors of in-hospital mortality.

## Data Availability

All data are available from the corresponding author on reasonable request.

## Abbreviations

IE: Infective endocarditis
MRSA: Methicillin-resistant *Staphylococcus aureus*
MSSA: Methicillin-sensitive *Staphylococcus aureus*
CNS: Coagulative-negative Staphylococcus
HAECK: Haemophilus, Aggregatibacter actinomycetemcomitans, Cardiobacterium hominis, *Eikenella corrodens*, and Kingella
